# PSMA PET in Active Surveillance: Initial Experiences and Future Directions

**DOI:** 10.3390/cancers18091376

**Published:** 2026-04-26

**Authors:** Jonathon Carll, Jordan Santucci, Sarah Whitty, Jacinta Bonaddio, Marlon Perera, Dixon Teck Sing Woon, Mark Frydenberg, Nathan Lawrentschuk

**Affiliations:** 1 EJ Whitten Prostate Cancer Research Centre, Epworth Healthcare, Melbourne, VIC 3052, Australia; 2 Department of Urology, The Royal Melbourne Hospital, Melbourne, VIC 3052, Australia; 3 Department of Surgery, University of Melbourne, Melbourne, VIC 3010, Australia; 4 Division of Cancer Surgery, Peter MacCallum Cancer Centre, Melbourne, VIC 3000, Australia; 5 Department of Urology, Cabrini Cancer Institute, Melbourne, VIC 3144, Australia; 6 Department of Urology, Queen Elizabeth II Jubilee Hospital, Brisbane, QLD 4108, Australia; 7 School of Translational Medicine, Monash University, Melbourne, VIC 3800, Australia; 8 Department of Urology, Monash Health, Melbourne, VIC 3168, Australia; 9 Department of Surgery, Austin Healthcare, Melbourne, VIC 3084, Australia

**Keywords:** prostate cancer, PSMA PET, active surveillance

## Abstract

**Background/Objectives**: We conducted an exploratory review of patients in our institutions who had undergone a PSMA PET/CT whilst on active surveillance, determining patient characteristics and trends. **Methods**: A retrospective cohort study of patients who were on active surveillance for low or favourable intermediate-risk prostate cancer and had a PSMA PET/CT done due to the presence of risk factors was performed. Risk factors that were an indication for PSMA PET/CT were: The presence of ISUP GG 2 disease, or ISUP GG1 disease with a PSA > 10 or a PI-RADS score of 4 or 5 on their MRI. **Results**: We identified 45 patients who underwent PSMA PET/CT whilst on active surveillance. Of these patients, 14 remained on active surveillance at the time of review, whilst 31 had progressed to definitive treatment. There was a significantly different distribution of PRIMARY scores between these two groups, although the average SUVMax was similar. In our practice, we found PSMA PET/CT to be particularly useful when performing confirmatory biopsies on patients who had normal MRI scans or were unable to go for the MRI at the time of diagnosis. **Conclusions**: PSMA PET/CT shows promise as a tool for active surveillance, particularly in men with risk factors for progressing to active treatment. However, our cohort was too small to draw definitive conclusions, and further research is needed.

## Simple Summary

We conducted a retrospective review of patients at our institutions who underwent PSMA PET/CT whilst on active surveillance for low or favourable intermediate-risk prostate cancer. This patient cohort was limited to patients with risk factors whilst on active surveillance, including low-volume ISUP grade group 2 disease, PI-RADS 4 or 5 lesions, or patients with ISUP GG1 disease and a PSA > 10. We identified 45 patients, of whom 31 left active surveillance and required definitive treatment. Between these two groups, there was no difference in SUVMax; however, patients who left active surveillance had a higher PRIMARY score. We also found PSMA PET/CT to be a useful tool when patients with negative or no MRI went for a repeat biopsy, allowing for an accurate targeting of lesions, leading to pathological upgrading.

## 1. Introduction

Active surveillance is a curative approach to low-risk and favourable intermediate-risk prostate cancer, which involves closely monitoring a patient with the goal of deferring or delaying significant interventions such as radical prostatectomy or radiotherapy [1]. This approach aims to minimise side effects and preserve the quality of life without compromising oncological outcomes [2]. Although there are different guidelines and protocols, the approach will typically involve regularly checking the patient’s PSA levels, along with a repeat digital rectal exam [3]. A key component of many modern active surveillance protocols is a repeat or confirmatory biopsy, conducted after initial diagnosis. This is important as core needle biopsies can under-sample or miss areas of higher-grade cancer [4,5], leading to an incorrect risk stratification of men who might not be otherwise suitable for active surveillance. Currently, major guidelines such as the EAU [6] and AUA [7,8] recommend a confirmatory biopsy for men within 12–18 months of starting active surveillance, if the patient did not undergo an MRI for their prostate prior to their initial biopsy. MRI of the prostate is becoming increasingly important in both the detection of prostate cancer and in active surveillance. MRI can reliably detect high-risk lesions and guide prostate biopsies. This has been proven to increase biopsy sensitivity and reduce the detection of lower-risk prostate cancer, which is often deemed clinically insignificant [9,10]. As such, MRI is becoming increasingly incorporated into many active surveillance protocols [3,11].

Recently, PSMA PET/CT has emerged as another revolutionary imaging technology in prostate cancer, although its potential role in active surveillance remains investigational. It is firmly established as the most accurate imaging modality for staging prostate cancer and detecting locally advanced and metastatic disease [12,13]. There is also a growing body of evidence that intra-prostatic PSMA uptake, as measured by SUVMax, is correlated with the presence of clinically significant prostate cancer (csPCa) [14]. As such, there has been much interest in utilising PSMA PET/CT as a tool for risk-stratification to further characterise prostate cancers that may have aggressive features [15]. This has led to ongoing research into applications for PSMA PET/CT beyond traditional staging into areas such as cancer detection and surveillance [16]. The PRIMARY study has demonstrated that the PSMA PET/CT is equivocal to mpMRI in detecting csPCa [17,18] and that combining both modalities can improve the accuracy of diagnosing csPCa [18]. This has generated great interest in whether PSMA PET/CT may be of use in an active surveillance algorithm as a potential non-invasive way to identify patients that may have been under-graded on their initial biopsy and are harbouring occult csPCa [19,20]. The possibility of misclassifying a prostate cancer as a low-risk or favourable intermediate-risk due to an inadequate biopsy presents a significant challenge when selecting patients for active surveillance [3,4]. As such, there has been great interest in incorporating medical imaging technologies, such as MRI, into active surveillance protocols to better detect patients harbouring higher-risk cancer [21].

So far, only a limited number of papers have assessed the utility of PSMA PET/CT in active surveillance. The largest study so far is the PASPoRT trial, which assessed 141 patients who underwent PSMA PET/CT during active surveillance and found a number needed to scan (NNS) of 11 to detect one patient who would be upgraded on repeat biopsy [22]. However, this trial had significant limitations, including a lack of central review of the pre-biopsy MRI and the fact that only 32% of the patients had a second biopsy based on PSMA PET/CT, making it uncertain how many patients would have had their cancers upgraded on a standard confirmatory biopsy without PSMA PET/CT targeting. This trial was able to successfully demonstrate that there are some patients who are initially misclassified at the time of diagnosis, who will have a higher-grade disease detected on PSMA PET/CT. This is an area of ongoing interest, with at least two other prospective trials running, designed to assess the utility of PSMA PET/CT in active surveillance [19,20]. However, to date, the published literature outside of the PASPoRT trial [22] has been limited to smaller series examining small cohorts, which have undergone PSMA PET/CT whilst on active surveillance [23,24]. Whilst insightful and reflective of real-world practice, these studies reflect local practice and are not generally applicable. There is currently a significant evidence gap between real-world practice and large ongoing trials, which are still running and have yet to publish results. Whilst we await results from larger perspective trials, we set out to assess our own local practice and contribute to the emerging evidence base and real-world usage of PSMA PET/CT in active surveillance.

In Australia, PSMA PET/CT is currently funded publicly through the Medicare Benefits Schedule (MBS) [25], where the government pays a rebate to a provider for patients to have initial staging of intermediate to high-risk prostate adenocarcinoma [25]. As such, public funding for patients suitable for PSMA PET/CT for active surveillance is limited to patients with intermediate-risk (including favourable intermediate-risk) diseases, such as those with ISUP GG2 or PSA > 10. Additionally, patients may pay out-of-pocket for PSMA PET/CT scans, or some public hospitals may publicly fund scans for select patients at the recommendation of senior clinicians. As such, the use of PSMA PET/CT in active surveillance is still in its infancy in Australia, with only small volumes of patients undergoing the scan as part of surveillance. We conducted a retrospective study on patients who underwent a PSMA PET/CT whilst on active surveillance at our institutions, aiming to explore and quantify our initial experiences within this cohort.

## 2. Materials and Methods

This is a retrospective multi-centre study performed from January 2022 until October 2025. All patients who underwent a PSMA PET/CT whilst on active surveillance for either low-risk or favourable intermediate-risk prostate cancer with a small volume of pattern 4 diseases were included in this study, with the exception of patients who were enrolled in other prospective clinical trials involving PSMA PET/CT, which were excluded. All patients underwent a PSMA PET/CT at the recommendation of a multi-disciplinary team (MDT). The MDT was a local board of urologists, radiologists, medical oncologists, and radiation oncologists who met weekly to review and discuss patient results to provide treatment recommendations for patients with genitourinary cancers. The MDT recommended PSMA PET/CT for patients on active surveillance that were deemed at high risk of failing AS due to the presence of one of the following risk factors: a PI-RADS 4 or 5 lesion on pre-biopsy MRI [26], a favourable intermediate-risk disease–GG1 disease with a PSA > 10 (either at time of biopsy or that progressed to >10 whilst on surveillance), or a GG2 disease suitable for active surveillance (<20% pattern 4, maximum 2 positive cores). Definitions of low and favourable intermediate-risk prostate cancer were as per the EAU risk groups [6]. All patients without a contraindication underwent MRI prior to the initial biopsy. Patients underwent a systemic biopsy with cognitive fusion targeting of any lesions present on the MRI with a PI-RADS score of 3 or greater. All PSMA PET/CT scans were performed using 18F DCFPYL tracers, with an injection-to-scanning time of 60 min as per the EANM and SNMI procedure guideline [27]. The CT component of the PSMA PET/CT was a low-dose non-contrast scan. SUVMax was reported on a per-lesion basis, along with the PRIMARY score of any underlying lesions. Reporting was done by specialist nuclear medicine physicians with experience in PSMA PET/CT at the local institution where the scan was performed. Patients were then recommended a repeat systemic biopsy that targeted any PSMA PET/CT-avid lesions (PRIMARY score of 3 or greater). The principal outcome assessed was the characteristics of the patients who failed active surveillance and the impact PSMA PET/CT had on patient management. The patient data that was collected included demographics, PSA levels, follow-up length, PSMA PET/CT scan results, biopsy grade group, whether the patient continued active surveillance, and what treatment modality they received if they progressed to active treatment. Statistical analysis was performed using JASP (Jeffreys’s Amazing Statistics Program version 0.95.1) [28]. Factors such as PSMA PET/CT SUVMax, PRIMARY score, and pre-biopsy PSA were assessed to see if they differed between patients who failed active surveillance and those who continued. Differences in patients who failed and continued active surveillance were compared using independent samples *t*-tests for continuous data and using the Mann–Whitney U test for ordinal data. Descriptive statistics of patient cohorts were also collected and assessed.

## 3. Results

Overall, 45 men were identified as having undergone a PSMA PET/CT whilst on active surveillance for low-risk or favourable intermediate-risk prostate cancer. The median age (IQR) was 64 years (57–70), with a median pre-biopsy PSA (IQR) of 5.5 ng/mL (4–7.7 ng/mL). At the time of review, the median follow-up time (IQR) for patients from the time of initial diagnosis by biopsy was 21.5 months (13–53). Twenty-three patients (51.1%) had GG1 disease, whilst 22 (48.9%) had GG2 disease. Thirty-four patients went on to have confirmatory biopsies whilst on active surveillance, whilst 11 did not receive a repeat biopsy. Table 1 provides an outline of the patient characteristics. Thirty-one patients left active surveillance, whilst 14 patients remained on active surveillance at the time of the review. Of the 31 patients who left active surveillance, 20 patients were recommended radical treatment after a repeat biopsy demonstrated either an increase in grade group or an increased volume of pattern 4 disease (>20% pattern 4 or more than two positive cores) deemed unsuitable for active surveillance. Two patients elected for definitive treatment due to personal preferences, four patients were recommended radical treatment due to PSA progression, placing them in the unfavourable intermediate-risk group despite stable volume of GG2 disease on biopsy, three patients had imaging (PSMA PET/CT or mpMRI) that demonstrated progression of their disease requiring definitive treatment, and two patients were transitioned to watchful waiting due to developing unrelated life-limiting illness. Patients who left active surveillance did not have a significant difference in the SUVMax on their PSMA PET/CT compared to patients who remained on active surveillance (*p* = 0.27). However, there was a difference in the distribution of PRIMARY scores between those that remained on surveillance and those that left active surveillance (*p* < 0.01) (Table 2).

Fourteen patients had negative MRI scans with a PI-RADS score of 1 or 2, with a further two patients being unable to undergo an MRI due to claustrophobia or incompatible pacemakers. Of this group of 16 patients, nine failed active surveillance (six upgraded on repeat biopsy, one with radiological progression, one with PSA progression, and one due to patient preference). Within this group of patients, 11 (69%) patients had a PRIMARY score of 3 or more, with a median SUVMax of 4.4. (Table 3).

An additional analysis was done of patients who failed active surveillance due to some form of disease progression (excluding patients who left surveillance due to preference, or due to co-existing morbidities causing them to progress to a watchful waiting approach) (Table 4). Within these patients, 74% had either a PRIMARY 4 or 5 lesion. Three patients were recommended active management based on the radiological findings from the PSMA PET/CT, two of whom had a SUVMax > 12, and one patient had local nodal disease identified on PSMA PET/CT (despite the core biopsy demonstrating < 10% of pattern 4 disease and a PSA of only 11.7 pre-biopsy). Within these three patients, all had GG2 disease with a small volume of pattern 4, and the decision to leave active surveillance was made entirely due to PSMA PET/CT findings alone. Of the four patients that failed due to PSA progression, three had a PRIMARY score of 4, which was considered as an additional supporting factor to recommend definitive treatment by the treating clinicians. Additionally, 20 patients failed due to pathological upgrading at the time of repeat biopsy after the PSMA PET/CT. Overall, the PSMA PET/CT was the primary driver behind the change to active management in 11% of patients and was a contributing factor in a significant number of other cases, although it is impossible to determine retrospectively how many of the patients would have been upgraded on repeat template biopsy regardless of a pre-biopsy PSMA PET/CT.

## 4. Discussion

Our results are consistent with prior studies that have demonstrated PSMA PET/CT is a useful tool in active surveillance. Although our cohort is too small to draw firm conclusions regarding the specific role PSMA PET/CT should play in active surveillance, it does validate the concept that it can be a useful tool. There was a difference in the PSMA PET/CT PRIMARY scores between groups of patients who discontinued active surveillance when compared to those who remained on active surveillance. Interestingly, within these two cohorts, there was no significant difference in SUVMax. Although these cohorts are too small to draw firm conclusions, it does suggest that a structured reporting system, such as a PRIMARY score, may be of more use in identifying higher-grade cancer requiring more treatment than SUVMax alone.

PSMA PET/CT was particularly useful in the cohort of patients who had a negative MRI prostate or were unable to undergo MRI. In this cohort, initial biopsies were done using a systematic template. However, 69% of this cohort had lesions on their PSMA PET/CT scan with a PRIMARY score of 3 or higher. The presence of these lesions allowed for more accurate targeting at the time of confirmatory biopsy, leading to pathological upgrading in 11 patients. This demonstrates how PSMA PET/CT scans can be used as an alternative to MRI when performing a cognitive fusion biopsy. Although further study is needed within this group of patients, the PSMA PET/CT shows promise as a tool to identify MRI-occult cancers, which may have been under sampled at the time of initial biopsy. Additionally, a small subgroup of patients had imaging findings on PSMA PET/CT that were decisive enough that they were recommended definitive treatment without a need for a further biopsy by their treating clinicians. This provides an insight into how PSMA PET/CT is already impacting patient management in the real world.

Active surveillance is an area of prostate cancer treatment that is rapidly evolving. There has been a trend towards guidelines recommending that patients with a low volume of Gleason pattern 4 disease are also suitable for active surveillance [6,7], which challenges the orthodoxy of active surveillance for patients with low-risk disease only [29]. With this paradigm change, there are new challenges for clinicians. Accurate targeting of the tumour on biopsy becomes crucial, as inaccurate targeting can cause the biopsy needle to only take a small glancing sample of the tumour, leading to an underestimation of the volume of pattern 4 disease. Therefore, within this group of patients, accurate targeting of the tumour is essential for proper risk stratification. This makes relying on an MRI for targeting problematic, as evidence shows that it can under-estimate tumour volume [30]. Subsequently, there may be a benefit to performing the PSMA PET/CT prior to confirmatory biopsy, as it may aid in detecting MRI-occult lesions, allowing for more accurate targeting on repeat biopsy.

Aside from the PASPoRT trial [22], at least two other papers have examined the use of PSMA PET/CT in active surveillance. Jain et al. retrospectively examined 30 patients with low-risk or favourable intermediate-risk prostate cancer who had undergone PSMA PET/CT after initial diagnosis [23]. In their cohort, 11 patients were recommended active surveillance, with only one moving towards active treatment with a prostatectomy. Pepe et al. performed PSMA PET/CT on 40 men with very-low risk prostate cancer, which had been on active surveillance [24]. They found PSMA PET/CT did not improve the detection of csPCa, but it did have a better diagnostic accuracy than mpMRI, and could have potentially spared 77% of men a repeat biopsy. These papers demonstrate that as PSMA PET/CT becomes more widely adopted, there are ongoing efforts to incorporate it into active surveillance protocols. Our data is similar to these prior efforts, as it demonstrates that PSMA PET/CT can provide additional clinical information when monitoring patients on active surveillance. This is reflective of growing local trends to utilise PSMA PET/CT in active surveillance. With increased public funding available for PSMA PET/CT [25], there is a growing population of patients on active surveillance in Australia who are eligible for funded PSMA PET/CT scans. As such, it has been adopted as a means to better risk-stratify patients on active surveillance, as well as a tool for targeting concerning lesions at the time of repeat biopsy. However, our cohort presents a more select group of patients that may be more likely to fail active surveillance, due to the presence of additional risk factors such as GG2 disease, PSA > 10, or a high volume of GG1 disease prior to starting active surveillance. This is evidenced by the fact that a high proportion of patients in our cohort (47.6%) ceased active surveillance within the reviewed timeframe. It may be that PSMA PET/CT is a more effective tool when selectively applied to cohorts that are more likely to fail active surveillance, although further research is needed.

Fortunately, there are ongoing efforts to better define the role of PSMA PET/CT in active surveillance. There are currently two high-powered prospective trials evaluating the use of PSMA PET/CT in large prospective cohorts of patients. Both the PIAS [20] and the CONFIRM [19,31] trials are assessing the use of PSMA PET/CT in patients with high-risk features on active surveillance. The data from these trials will allow for a further assessment of whether PSMA PET/CT has a role in surveilling these patients, building on local experience and hopefully allowing for the development of formal surveillance protocols incorporating PSMA PET/CT. This demonstrates that the ongoing direction of research is focused on patients with risk factors for failing active surveillance, rather than patients with low-volume GG1 disease or very-low risk prostate cancer. With both these trials underway, there should be high-quality evidence in the near future determining if there is a role for PSMA PET/CT in active surveillance.

## 5. Limitations

This study is limited by its retrospective design and small cohort of patients who were identified and met the inclusion criteria. As such, our results should be considered as an exploratory basis to launch further research. It is also limited in the variable and non-standard follow-up period, which is highly variable between patients at the time of review. Although it provides an insight into local practice and experience with using PSMA PET/CT in active surveillance, it is not sufficiently powered to draw firm conclusions from. The strengths of this study are the selection and identification of a higher-risk cohort of patients that may be more likely to benefit from PSMA PET/CT on active surveillance and the central review of imaging by the MDT. However, this is also another limitation, as this selection bias makes our results less applicable to populations undergoing active surveillance without these risk factors.

## 6. Conclusions

In our exploratory review, we were able to identify a small volume of patients who underwent PSMA PET/CT whilst on active surveillance at the recommendation of the MDT. Our cohort included only patients with high-risk features who we thought were more likely to fail active surveillance. Unfortunately, we did not have a large enough volume of patients to draw any firm conclusions. However, the PRIMARY score on PSMA PET/CT was different amongst patients who left active surveillance, and 57% of the patients who were scanned demonstrated a concerning lesion with a PRIMARY score of 4 or 5. This information was clinically useful, as it allowed for targeting of these lesions at the time of confirmatory biopsy. Ultimately, 31/45 patients proceeded to require definitive treatment, although within the timeframe assessed, not all patients with a PRIMARY score of 4 or more required definitive treatment. Longer periods of follow-up will be required to determine the predictive power of PSMA PET/CT to identify which patients are going to fail active surveillance. Our initial experiences demonstrate that in select patients, PSMA PET/CT may be of use in active surveillance and can be an effective tool to guide confirmatory biopsies. This is an ongoing area of investigation, with larger trials underway to determine the exact role PSMA PET/CT may play in active surveillance.

## Figures and Tables

**Table 1:** The patient characteristics.

Characteristic	Patients (n = 45)
**Age (years), median (IQR)**	64 (57–70)
**Pre-biopsy PSA (ng/mL), median (IQR)**	5.5 (4.0–7.7)
**Length of follow-up (months), median (IQR)**	21.5 (13–53)
**MRI PI-RADS score, n (%)**	
1	2 (4.4)
2	12 (26.7)
3	2 (6.7)
4	22 (48.9)
5	4 (8.9)
No MRI	2 (4.4)
**Initial biopsy grade group, n (%)**	
1	23 (51.1)
2	22 (48.9)
**Time on AS until PSMA PET/CT (months), median (IQR)**	13 (2–36)
**PSMA PET/CT SUVMax, median (IQR)**	6.25 (3.9–10.7)
**PRIMARY score, n (%)**	
1	8 (17.8)
2	3 (6.7)
3	7 (15.6)
4	18 (40.0)
5	9 (20.0)
**Indication(s) for PSMA PET/CT, n (%)**	
PI-RADS 4/5 lesion present	11 (52.4)
Grade group 1 PSA greater than 10	2 (9.5)
Presence of a small percentage of pattern 4	11 (52.4)
**Stopped Active Surveillance, n (%)**	31 (64.5)
PSA progression	4 (8.9)
Radiological progression	3 (6.7)
Upgraded GG on repeat biopsy	20 (44.4)
Patient preference	2 (4.4)
Transitioned to watchful waiting	2 (4.4)
**Months ceased active surveillance, median (IQR)**	24 (13–49)
**Final treatment choice, n (%)**	
Radiation	6 (13.3)
Surgery	20 (44.4)
Watchful waiting	2 (4.4)
Lost to follow-up	1 (2.2)
Continued active surveillance	16 (35.6)

**Table 2:** Comparison between patients who failed and continued active surveillance.

	Failed AS (n = 31)	Continued AS (n = 10)	*p* Value
PRIMARY Score,		data	
1	4	4	
2	1	2	**0.01** (Mann–Whitney U)
3	2	5	
4	15	3	
5	9	0	
SUVMax, median (IQR)	7.0 (4.1–12.4)	5.4 (3.6–7.0)	0.27 (*t*-test)
Pre-Biopsy PSA, median (IQR)	5.4 (4.5–7.6)	6.0 (3.1–8.6)	0.2 (*t*-test)
Length of follow-up (Months), median (IQR)	22.5 (13.5–18.5)	18.5 (10.3–69.8)	0.38 (*t*-test)

**Table 3:** Patients with negative or no MRI.

Characteristic	Patients (n = 16)
**Age (years), median (IQR)**	58.5 (49.8–67.25)
**Pre-biopsy PSA (ng/mL), median (IQR)**	5.6 (0.6–10.5)
**MRI PI-RADS, n (%)**	
1	2 (12.5)
2	12 (75)
No MRI	2 (12.5)
**Initial biopsy grade group, n (%)**	
1	11 (68.8)
2	5 (31.2)
**PSMA PET/CT SUVMax, median (IQR)**	4.4 (0.8–8.0)
**PRIMARY score, n (%)**	
1	4 (25)
2	1 (6.3)
3	4 (25)
4	5 (31.3)
5	2 (12.5)
**Stopped active surveillance, n (%)**	9 (56.2)
PSA progression	1 (6.3)
Upgraded GG on repeat biopsy	6 (37.5)
Patient preference	1 (6.3)
Radiological progression	1 (6.3)

**Table 4:** Patients who failed active surveillance due to disease progression.

Characteristic	Patients (n = 27)
**Initial biopsy grade group, n (%)**	
1	14 (51.9%)
2	13 (48.1%)
**PSMA PET/CT SUVMax, median (IQR)**	7 (3.8–12)
**PRIMARY score, n (%)**	
**1**	4 (14.8%)
**2**	1 (3.7%)
**3**	2 (7.4%)
**4**	12 (44.4%)
**5**	8 (29.6%)
**PI-RADS score, n (%)**	
**1**	2 (7.4%)
**2**	5 (18.5%)
**3**	0 (0%)
**4**	15 (55.6%)
**5**	4 (14.8%)
**No MRI**	1 (3.7%)

## Data Availability

The raw data supporting the conclusions of this article will be made available by the authors upon reasonable request.

## Abbreviations

The following abbreviations are used in this manuscript:

PSMA	Prostate Specific Membrane Antigen
PET	Positron Emission Tomography
MRI	Magnetic Resonance Imaging
SUVMax	Maximum Standardised Uptake Value
PSA	Prostate Specific Antigen
EAU	European Association of Urology
AUA	American Urological Association
csPCA	Clinically Significant Prostate Cancer
GG	Grade Group
MDT	Multi-Disciplinary Team
PI-RADS	Prostate Imaging and Data Reporting System
ISUP	International Society of Urological Pathology
AS	Active Surveillance
